# Taking Stock of Qualitative Methods of Evaluation: A Study of Practices and Quality Criteria

**DOI:** 10.1177/0193841X251370426

**Published:** 2025-08-28

**Authors:** Thilo Bodenstein, Achim Kemmerling

**Affiliations:** 1 Department of Public Policy, 47797 Central European University, Vienna, Austria; 2 Willy Brandt School of Public Policy, 38877 University of Erfurt, Erfurt, Germany

**Keywords:** qualitative methods, evaluation standards, content analysis, methodology, systematic review

## Abstract

Research on evaluation has mapped the landscape of quantitative evaluation methods. There are far fewer overviews for qualitative methods of evaluation. We present a review of scholarly articles from five widely read evaluation research journals, examining the types of methods used and the transparency of their quality criteria. We briefly look at a large sample of 1070 articles and then randomly select 50 for in-depth study. We document a remarkable variety of qualitative methods, but some stand out: Case studies and stakeholder analysis, often combined with interview techniques. Articles rarely define and conceptualize their methods explicitly. This is understandable from a practical point of view, but it can make it difficult to critically interrogate findings and build systematic knowledge. Finally, we find that the transparency of qualitative criteria required in the literature is not always sufficient, which can hinder the synthesis of results.

## Introduction

Qualitative evaluation designs are a powerful tool for analyzing processes in projects, programs and policies (Filstead, 1981). Their strength lies in analyzing interpretations of policy, incorporating the policy context and uncovering complex causal relationships (Maxwell, 2020). Qualitative evaluation is also increasingly used in the context of evidence-based policymaking. For example, Barnow et al. (2024) show how qualitative designs are particularly suited to triangulating data and exploring causal mechanisms in the area of mixed methods evaluation designs in impact studies. Despite the potential of qualitative evaluation designs, their results are also seen as ambiguous, more suitable for formulating hypotheses than for testing them, or more appropriate for small and local evaluations than for larger and more complex programs. They are often seen as biased or producing results that are difficult to generalize (Henry, 2015; Mackieson et al., 2019).

This is a problem because evaluations are not an end in themselves, but play a central role in the formulation of evidence-based policy and related forms of policy learning (Howlett, 2012; Sanderson, 2002). However, the contribution of evaluations to policy learning is only possible if evaluations adhere to transparent and comprehensible standards of causal inference or dissemination readiness (Buckley et al., 2020), which can be used to improve technical learning and knowledge utilization in the policy process. Systematic reviews and critical appraisals, which synthesize the state of knowledge and thus strengthen analytical capacity, are key tools in this regard. The extent to which qualitative evaluations should play a role in critical appraisal also depends on adherence to with reporting standards, as this is the only way to assess the quality of studies.

For example, Carroll et al. (2012), show that explicit specification of standards is a good measure of the methodological quality of a study and argue that qualitative evaluations should be excluded if reporting standards are not met. In a sensitivity analysis of 19 qualitative syntheses, they conclude that half of the qualitative studies had poor reporting standards and that poorly reported studies contributed little to the overall results, whereas only qualitative reports with high reporting standards drove the themes of the synthesis. While Verhage and Boels (2017) caution against excluding inadequately reported studies from scoping reviews, they still find 18 out of 31 qualitative studies in their review to be inadequately reported and call for adequate reporting of minimum information on methodology in qualitative studies. Walsh et al. (2020) find that of 197 studies in nursing journals analyzed, the quality of reporting was moderate (57%) or poor (38%) and argue for clear and transparent reporting of qualitative research to help counteract negative perceptions of qualitative research and improve its relevance and transferability.

The contribution of qualitative approaches to knowledge utilization and policy learning therefore depends on the extent to which they communicate credibility techniques in the production and analysis of empirical evidence (Anastas, 2004; Liao & Hitchcock, 2018). Professional association handbooks have long called for quality of inference and empirical evidence. Examples include the American Evaluation Association, the Canadian Evaluation Society (Yarbrough et al., 2011), or the various national evaluation associations in Europe (e.g. European Evaluation Society). Criteria for the credibility of qualitative evaluation thus exist and the primary question is whether they are also applied and reported in evaluations (Macklin & Gullickson, 2022).

A complication arises from to the methodological diversity of qualitative evaluations, which makes it difficult to synthesize findings based on heterogeneous methods with heterogeneous purposes (Lawarée et al., 2020). For example, qualitative approaches compromise a multitude of different ontological and epistemological positions. As Patton (2015, p. 164) puts it: “Qualitative inquiry is not a single, monolithic approach to research and evaluation.”

In this contribution, we are interested in two related objectives in this context. We examine the extent to which qualitative evaluations follow the methodological recommendations found in the literature on qualitative research methods, and how they report on these standards. A common foundation of qualitative approaches and common practices of reporting thus play a role in practical and academic discussions of evaluation. The findings of qualitative evaluations, in turn, depend on the approaches and methods that are common and accepted in the field. For this reason, we also document the methods, instruments, and empirical strategies used, since the quality standards reported refer to them. In addition to documenting evaluation methods as they are published, this also helps us to identify patterns across different methods.

In the following, we analyze in several steps evaluations that primarily use qualitative methods and were published in scientific journals between 2015 and 2019. First, we briefly look at all 1070 eligible articles. We examine those with a simple content analysis to show the variety of qualitative approaches and methods used. In a second step, we draw a sample of 50 articles, which we code manually. We follow established reporting and quality criteria for qualitative research.

In interpreting the findings, we proceed in three steps: First, we look at the variety of methods, approaches, and data sources used. Then we show the extent to which the articles provide insights into methodological standards. Finally, we relate both types of information, to see whether some articles using certain types of methods also report certain types of standards. As expected, the results show a wide range of qualitative evaluation approaches, but they often converge on relatively few designs and empirical sources: Case studies are the most common evaluation design; stakeholder analysis and community analysis are two of the most important categories of evaluation, while interviews tend to be the most important source of information. When it comes to reporting on quality criteria, there seems to be no common practice yet. For example, relatively few articles discuss the role of the evaluator in evaluation, let alone a deeper reflection on positionality, and the role of the evaluator in the evaluation context. While this is understandable from a practical point of view, e.g. due to time constraints, it represents a missed opportunity for a more systematic inquiry. We also find few systematic differences between the methods. For instance, case studies are not much more likely to report transferability than other types of evaluation designs, although case studies would naturally lend themselves to such discussions.

In the next section, we discuss criteria and reporting standards in qualitative research and evaluation. In the third section, we explain our strategy for selecting and coding our smaller sample. The fourth section presents the results. In the final section, we discuss the implications of our findings not only for the narrow field of qualitative methods of evaluation, but also for building knowledge and leading to policy learning in more general.

## Methods and Reporting Practices of Evaluation

The field of qualitative evaluation methods is diverse, so our contribution can hardly do justice to all the varieties and purposes of evaluation, but there are excellent overviews for qualitative evaluation methods (Bamberger et al., 2009; Befani, 2020; Donaldson & Lipsey, 2006; Donaldson & Scriven, 2003; Maldonado Trujillo & Pérez Yarahuán, 2015; Patton, 2015). We will talk more about the diversity of methods in our section on how we coded the articles, but one source of this diversity is worth highlighting. One important difference is the epistemology on which the qualitative methods are based, which is relevant to the question of what quality criteria should be used. While many qualitative approaches such as process tracing often follow a positivist logic, others combine insights from positivist and constructivist logics (e.g. Pawson & Tilley, 1997), or are firmly ground in interpretative-hermeneutic perspectives (e.g. Jones et al., 2016).^1^

As a result of these different theoretical traditions, few studies have mapped the diversity of the field. There are, however, excellent textbooks both on evaluation research (Patton, 2015; Shaw, 1999) as well as qualitative research methods (Creswell, 2007; Denzin & Lincoln, 2018; Maxwell, 2013) which provide tools for categorizing different approaches. There is also a vibrant discussion of research on evaluation that echoes many of the themes we develop below (Apgar et al., 2024; Azzam, 2011; Christie & Fleischer, 2010; Coryn et al., 2017; Lawarée et al., 2020; Leininger & Schiller, 2024; Miller, 2010; Smith, 1993; Teasdale, 2021; Teasdale et al., 2023).

Against the background of methodological diversity in qualitative evaluation, there have been calls for professionalization (Picciotto, 2011; United Nations Evaluation Group, 2016), and for common reporting practices in evaluation reports (Carroll et al., 2012; Miller, 2010; Montrosse-Moorhead & Griffith, 2017). While some of these standards already exist, convergence towards common labelling remains a challenge, as there are multiple assessment tools for different subject areas (Majid & Vanstone, 2018). In the remainder of this section, we briefly consider the difficulties of developing common quality criteria for qualitative social research and evaluation and review existing catalogues for common standards in the field of qualitative evaluation.

In the field of evaluation practice, Macklin and Gullickson (2022) show the broad spectrum of conceptualizations of the term “validity”. Depending on the underlying research paradigm, validity can be conceptualized as internal or external validity, or as translational or interpretive validity, to name just a few approaches. Validity can also be conceptualized more narrowly in terms of research design, data sources and sampling, and measurement, or more broadly, to include the preparation, reporting of results, and evaluation use. Again, scholars find a wide variety of concepts in their synthesis of the corresponding literature.

If we want to examine the quality criteria used in qualitative evaluations, we cannot work with the criteria of the positivist approach alone. Criteria exported from quantitative, positivist research are not sufficient to account for most problems in qualitative evaluation research. However, qualitative researchers have proposed alternative criteria (Jacobs et al., 2021, pp. 186–187). One example is “trustworthiness” (Guba & Lincoln, 1989; Lincoln, 2005; Schwartz-Shea & Yanow, 2012, pp. 91–114) of the research findings instead of validity and replicability. The methodological literature offers a number of benchmarks in this regard, some of which are specific to certain approaches (cf. Creswell, 2007, p. 203).

Taking inspiration from Apgar et al. (2024) and others, the following grouped characteristics are crucial for the credibility or trustworthiness of qualitative research as a minimum requirement: Reflexivity, confirmability and transferability. First, reflexivity, and, relatedly, positionality, is a fundamental criterion of qualitative research (Alvesson & Sköldberg, 2018; Schwartz-Shea & Yanow, 2012, pp. 99–104). Qualitative research acknowledges the influence of the researcher on the participants; researchers are part of the “field”, rather than outside observers. This perspective is important for evaluation, especially for participatory forms of evaluation. It is also important for readers of evaluation research to better understand the role the evaluator plays in the evaluation.

The second criterion is confirmability, a standard closely related to transparency. Since qualitative methods have to reconcile various sensitive issues such as protecting sources, building trust, and honoring contextual information, transparency of research is secured by confirmable procedures, such as “member checking” (Schwartz-Shea & Yanow, 2012, p. 106), a detailed description of the process of analysis, coding procedures and inter-coder agreement (Creswell, 2007, pp. 209–210). Similar to Carroll et al. (2012), however, we consider the justification of the choice of methods and the selection of data sources to be important for a transparent approach.

The final criterion is the transferability of results (Schwartz-Shea & Yanow, 2012, pp. 47–48), which is often not the central aim of qualitative research and evaluation. Still, interrogating in how far context-dependent knowledge can travel is an important issue, as evaluation research aims to highlight aspects that are important beyond the specific intervention. In synthetic or systematic reviews this criterium plays a huge role. We are therefore interested in the transferability of the results to other cases with a similar context (Guba & Lincoln, 1989; Lincoln, 2005). To give just one example: a “thick description” of the case under evaluation might help to transfer the findings of the case to a broader field of similar cases (Schwartz-Shea & Yanow, 2012, p. 145), or also a discussion of the contextual conditions under which the results are transferable. All in all, we consider reflexivity, confirmability and transferability as minimum requirements for the trustworthiness of qualitative evaluations.

Table 1 provides our stylized overview of the methodological debate on quality criteria.^2^ Note that we do not argue that these standards should apply exclusively to quantitative or qualitative approaches. Nor do we wish to draw strong analogies between a standard such as objectivity on the quantitative side, and reflexivity on the qualitative side. Nevertheless, these three rather stylized methodological criteria are well established among qualitative methodologists and experts in evaluation methodology. With these clarifications in mind, Table 1 (right side) will inform most of our coding for the quality criteria in the rest of the paper.

**Table 1.** Overview of Qualitative Criteria in Social Sciences

Quantitative	Qualitative
Objectivity/ Inter-subjectivity	Reflexivity/ Positionality/(In-)Dependence
Validity, reliability, replicability	Transparency/ Confirmability
Generalizability	Transferability

*Note.* Own compilation based on the sources above.

## Selection of Articles and Coding Procedure

Qualitative researchers emphasize the need to be transparent about one’s own positionality as well as initial failures and a learning in the research process (Jacobs et al., 2021). We therefore begin with some reflections on our own independence and positionality. We are both academic researchers with practical experience in evaluation, but we are not involved in any organization or ongoing evaluation activity. In particular, we have no direct relationship with any of the coded articles analyzed below. Given our personal research experience, we may both have a bias towards quantitative evaluation methods and the positivist paradigm, as our own research often uses quantitative studies. Therefore, we try to be as transparent as possible about the following research methodology and our trial-and-error approach to making sense of the information.

Our selection of articles has two goals. First, it should cover a wide range of comparable qualitative evaluations, and second, it should be accessible to a broad readership. Thus, we consider articles published in peer-reviewed journals because they are read by a wide audience, are a relatively homogeneous group, and often focus on methodological issues. Similar to Christie and Fleischer (2010), Coryn et al. (2017) and Teasdale et al. (2023), we focus on major, general evaluations journals. This also implies that we do not include grey literature, which often contains more information such as methodological appendices.

To select the journals, we first looked at the full list offered by the Social Sciences Citation Index, especially those listed as “social science interdisciplinary” and “evaluation” journals. We have avoided specific journals that focus only on evaluation in specific areas such as health, education, etc. Our final list includes *American Journal of Evaluation*, *Canadian Journal of Program Evaluation*, *Evaluation*, *Evaluation and Program Planning* and *Evaluation Review*. For methodological reasons, we chose a period of 5 years (2015–19), resulting in a total of 1070 articles after the deletion of 80 ineligible studies (see Appendix 1).^3^

**Figure 1.**
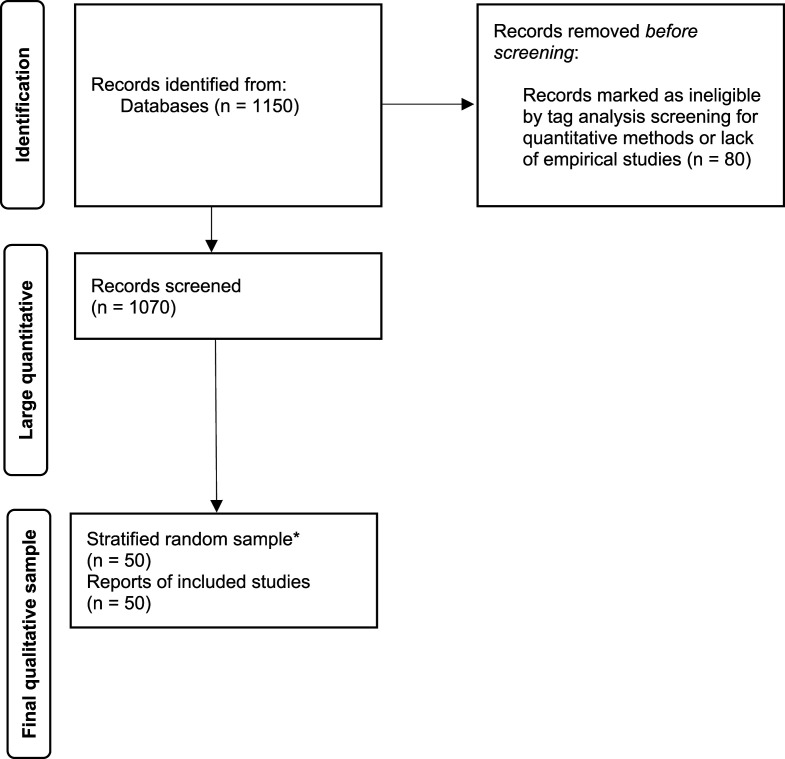
PRISMA Flow Chart for Identification of Studies via Databases and Registers. Flow Diagram Adopted From Page et al. (2021). * Based on New Random Sample Stratified by Year

The flow diagram in Figure 1 illustrates our approach. In our first attempt, we checked all articles for qualitative evaluations, using the tags (and keywords) provided by the journals as a first filter. In particular, we excluded studies that were theoretical contributions without reference to any kind of cases or empirics. This first selection resulted in 1070 articles, the total number of qualitative articles in the selected journals for the period under review. With these articles, we did some simple keyword searches to get a sense of the overall collection of articles. Although this quantitative content analysis is rather basic (see below), it does give some indication of the relationships between all the articles and the articles that were finally selected and coded.

We tested our initial coding scheme with a random sample of 95 papers. We coded these articles according to a simple coding scheme that gives us a first overview of the distribution of the articles in relation to the categories listed in Appendix 2. We coded 54 articles separately, and 41 articles jointly. We then checked the jointly coded articles for inter-coder reliability, which resulted low at 52.2% (Krippendorff, 2011, pp. 211–256). After discussing and correcting the discrepancies in our coding strategies, we decided to adapt our coding strategy and to use a two-stage deliberative coding procedure (e.g. Hak & Bernts, 1996; Saldaña, 2021).

In our final approach we started with a new stratified random selection of 10 articles from each year to reach our final sample of 50 articles from 2015–19. The random selection of articles mainly serves to minimize our own bias in terms of topics and methods. In the first round, we coded the articles separately. In the second round, we discussed each article and revised our coding. In very few cases, we could not agree on a common coding, either because we had different impressions or because the articles were not clear enough. However, for the vast majority of the codings, we agreed after the second stage, resulting in a final intercoder reliability of 96.4% (Appendix 3). This high result is not surprising given the deliberative approach. It simply documents the change in our own research strategy and gives some indication of where problems may have arisen in our coding strategy.

## Results

### Sample Characteristics and Types of Methods

Before looking at the results of the small sample of 50 articles, we present some comparative information for the initial, larger sample of 1070 articles and compare it to the smaller, final sample (see list of articles in Appendix 9). The comparison should be treated with caution, as the coding of the large sample was done by simple keyword search. As is well known (e.g. Krippendorff, 2011), simple (lexicographic) keyword searches have validity issues. For instance, our keyword searches resulted in a many missing observations (see below). Nevertheless, comparing the keyword searches with our deliberative coding reveals interesting cross-cutting forms of evidence and improves the transferability of the overall findings. When we compare the distribution of the studies’ evaluands across sectors, we see that the sectoral distribution is relatively similar between the two samples (Table 2). This suggests that the smaller sample of 50 articles is not very different from all articles between 2015 and 2019.

**Table 2.** Sectoral Distribution of Coded Articles

	50 articles		All articles	
Sector	Frequency	Percent	Frequency	Percent
Development	6	8	115	11
Education	12	17	97	9
Social & labor	8	11	137	13
Economic	4	6	32	3
Health	30	42	258	24
Administration	5	7	41	4
Infrastructure	1	1	8	1
Crime/Security	1	1	12	1
Other	4	3	358	34

### Qualitative Methods, Designs and Sources

We briefly document the enormous diversity of qualitative evaluation methods and their contexts. As discussed above, we have collected information on evaluation context, and the types of evaluation methods. We highlight some of these findings for the small sample of 50 articles. First, we look at the main categories of evaluation from which we drew inspiration (Kusters, 2011; Mathison, 2005; Newcomer et al., 2015; Patton, 2015; Shaw, 1999; Shaw et al., 2006). It is difficult to draw clear lines between these categories. We recognized this in our own coding process. For this reason, Table 3 presents two types of frequencies. One shows the initial codes we assigned. The idea behind the first codes is that it is the most intuitive choice of both coders, the category that first came to mind while reading the article. We contrast this with all codes given, in those cases where we allowed for multiple codes. For example, “Evaluation categories” is such a case of multiple coding. Regardless of whether we use the first or all codes, we find that stakeholder analysis, broadly defined, is by far the most common category. The next categories are developmental and community-based evaluations, followed by mixed methods and evaluations that include some form of theory of change or logic model.

**Table 3.** Evaluation Categories

	1st Codes		All Codes	
	Frequency	Percent	Frequency	Percent
Stakeholder analysis	17	41	23	32
Community approach	5	12	6	8
Realist evaluation	3	7	3	4
Contribution analysis	1	2	2	3
Participatory approach	2	5	9	13
Developmental evaluation	4	10	8	11
Process tracing	1	2	1	1
Mixed methods	3	7	7	10
Theory of change/ logic model	3	7	9	13
Network analysis	2	5	2	3
Experimental	0	0	2	3
Total	41	100	72	100

Some of these categories co-occur with each other relatively frequently (Appendix 4). For instance, community and participatory approaches often coincide, as do participatory approaches and stakeholder analysis. Not surprisingly, realist evaluations often include a theory of change. Others are more orthogonal, meaning that they do not occur as often with other categories, especially the less common ones. Here are some examples of less common combinations: Millett et al. (2016) combine a theory of change with developmental evaluation; Suiter (2017) combines realist and community evaluation; Chen (2017) combines stakeholder analysis with a quantitative approach in an importance-performance analysis; Frye et al. (2017) combine community analysis, realist evaluation, and theory of change with a quasi-experimental approach; and Koleros et al. (2016) use a mixed-methods approach with community and contribution analysis and a theory of change.

In general, we find that the different categories and approaches are very diverse, but some categories are clearly more dominant (see Table 3). We must also emphasize that larger categories such as stakeholder analysis hide a great deal of heterogeneity within the category (see below).

The diversity of qualitative methods is also evident in Table 4, which lists the different empirical methods of information gathering that we have coded following Patton (2015). All articles contained interpretable information on the main empirical sources used. Again, the table shows both first codes and all codes. The most common category here is “interviews”. This is not very surprising, as stakeholder, community, and participatory approaches dominate the previous table. The reporting on the exact nature of these interviews varied greatly from article to article, so it is difficult to make a summary assessment. However, there are clearly major differences in the interview methods used in the sampled articles.

**Table 4.** Data Sources

	1st Codes		All Codes	
	Frequency	Percent	Frequency	Percent
Survey	11	22	12	12
Interviews	20	40	28	29
Focus groups	3	6	18	18
Documents	15	30	26	27
Participatory workshops	0	0	10	10
Direct observations	1	2	4	4
Total	50	100	98	100

Documents, broadly defined, are the second most important category, but even here there is considerable heterogeneity. Often “documents” refers to official documents (such as policies, laws, regulations), but the category also includes field notes, protocols, and other types of written records produced during the evaluation. Some categories were less common than we expected. For example, direct observations are rare. Sometimes we also found it difficult to distinguish between different categories – for instance, focus groups and participatory workshops were sometimes indistinguishable.

Further results on the different types of evaluation methods are reported in the appendices. The most common research design we could identify was the case study method (Appendix 5). Like “stakeholder analysis”, “case study” is a very broad category. Very few articles explicitly defined the term “case” or discussed in detail issues related to the design of case study research. It was often implicit what the universe of cases should be and how the case under study relates to that universe.

Most articles included some form of program or project evaluation, but there were also many studies that focused on research on evaluation. Explicit meta-studies (meta-narratives, meta-analyses etc.) were comparatively rare, as we excluded most quantitative evaluations (Appendix 6). Three quarters of all articles followed some form of the positivist paradigm. Constructivist and interpretative approaches were relatively rare (Appendix 7). Similarly, “causal analysis” was the most common category, but closely followed by descriptive and explorative studies (Appendix 8).

In this overview, we see that three approaches and three methods are predominantly chosen to generate qualitative evaluation evidence. Stakeholder analysis, community approach, and developmental evaluation account for 63% of the main approaches chosen. The most important research design is the case study. Data collection is mainly through interviews, documents and surveys (92%). This is interesting in that qualitative evaluations have a wide range of approaches and methods at their disposal. In practice, however, they focus on far fewer techniques, almost as if there were an informal consensus on how to achieve qualitative results. At the same time, many studies take a more holistic view, combining multiple approaches, designs, and data sources. With this information about the types of methods, we can now turn to the reporting criteria to see if there are differences between these methods, broadly defined.

### Reporting of Quality Criteria

In this section, we present the results for articles coded using the credibility criteria listed in Table 1, supplemented with some categories from the Checklist for Evaluation-Specific Standards (CHESS) (Montrosse-Moorhead & Griffith, 2017), which is a comprehensive catalog of criteria that provides a common quality base across disciplines and methods.

Let us begin with perhaps the most important category – the independence of the evaluator, which is the first step in discussing reflexivity and positionality. Although explicit discussion of independence was rare, we tried as best as we could to code information about the authors of the articles to assess their role in the evaluation. We distinguished three cases: In the first case, the authors are both evaluators and policy makers. In the latter case, we mean that they are also responsible for the intervention itself. This was the case in almost 30% of the articles. This form of (in)dependence predominates in participatory or developmental evaluations, for example, when representatives of health organizations are part of the evaluation team and part of the team that wrote the research article. More common, however, is the situation where the authors are also the evaluators but are not responsible for the intervention itself, which is the case in 56% of the articles. This is perhaps the classic case of a formally independent evaluation.

Of course, we know very little about the detailed social background of the evaluators and how close they are to those responsible for the intervention. For us, independence was expressed only by the method of exclusion: the authors did not appear to have been involved in the original design of the intervention. Finally, in only 10% of the articles were the authors different from the evaluators and policy makers. This was the case when we coded meta-studies, i.e. authors who reviewed evaluations of others. Transparency about independence helps readers of the evaluation study better assess the quality of the findings, as shown by Sturges (2015):

“I became involved in College Now (CN) when I was recruited during the program’s third year by the firm hired to perform its evaluation. One of my first evaluations, I oversaw classroom observations, interviews with students and teachers, and AP course plans and materials. At the same time, I was conducting a qualitative study on reform at two CN schools. (...) The simultaneity of projects provided me a unique insider–outsider perspective on CN and helped me reflect on my responsibilities as a junior evaluator. (p.462).”

**Figure 2.**
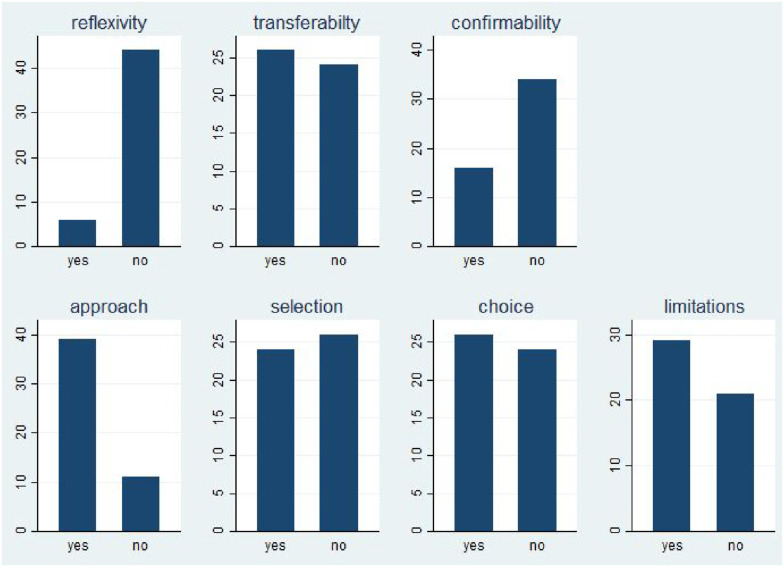
Frequencies of Standards Reported. Note: Own Graph on Basis of Coding for 50 Articles

We also looked more closely at reflexivity and positionality. In our coding, “reflexivity and positionality” was present when authors explicitly described their own role as well as potential biases, subjective interpretations, and reflexive relationships. Figure 2 shows the percentage frequencies of simple yes and no dummy questions when we considered a quality criterion to be explicitly discussed (1) or not (0). “Reflexivity” is the first bar in Figure 2 and shows that only a small percentage of articles address reflexivity and positionality. Similarly, a keyword search for reflexivity in the large sample of 1070 returns only two direct hits. And yet, there are exemplary accounts of reflexivity, such as Siebert and Myles’s (2019) deliberate approach to the implications of their own role as external evaluators:

“Our initial failure to reconstruct a programme theory that provided an accurate representation of the programme made us reflect on our role as evaluators. How much direction do we as evaluators provide when this becomes apparent? Should we reconstruct the theory for the programme as we perceive it, or should we focus on using methods that will facilitate a process that will enable stakeholders to do so themselves? (...) It was these questions and reflections that made us decide to find a way to remove misrepresentation and be assured that the process of reconstructing the programme theory was both transparent and representative of stakeholders’ collective logic. (p.471)”

Of course, not all types of evaluation methods require a deeper engagement with reflexivity and positionality. Nevertheless, we think it is worth considering whether mainstreaming such considerations might not be an important aspect of all evaluation research. Qualitative researchers are very sensitive to such issues and could only strengthen their own contributions by being open about their positionality. Although some might see this as weakening their own evidence,^4^ such a concern should be irrelevant in an academic context. Instead, if reflections on reflexivity and positionality become part of the reporting routine, this could greatly facilitate readers’ understanding of the evaluation context and evaluation research.

The next major quality criterion is transferability (Figure 2, first row). According to our coding, nearly 50% of all articles discuss the transferability of findings to other cases, domains, fields, or interventions. What exactly counts as transferability is, of course, context-dependent, but it is extremely helpful for a reader of such articles to know the author’s assessment of which parts of the general lessons would be transferable to other applications. Note that we coded transferability as “1” even if the authors explicitly mention that generalizations from the case are difficult or even impossible for specific reasons.

Often it is useful to dismiss the idea of transferability, but in most cases explicit discussion is helpful, especially when authors refer to their work as a “case study”. Contrary to our expectations, those studies for which we coded the research design category as case study did not have a significantly higher proportion of discussions of transferability. About 50% of all case studies discussed the transferability of findings (see below). In our view, this is a missed opportunity, as an open discussion of authors’ views on transferability would be very helpful. It would give us the opportunity to synthesize the results and produce systematic reviews based on many cases. Nevertheless, there are interesting examples of how to communicate the transferability of results. For example, the role of context in the transferability of results is also highlighted by Nielsen et al. (2019):

“A strength of this study is the focus on the provider perspective of the implementation processes and the contextual descriptions of a successful school-based programme tripling the amount of PE. (…) This potentially increases practitioners and decision-makers ability to assess the programme in relation to their individual context (…) – ultimately strengthening the transferability of the programme and the strategies used to secure the implementation of additional PE or PA in a school context. (p. 7).”

The importance of confirmability as a quality criterion is highlighted in Table 1. Of course, such a criterion is not easy to code. One thing we coded directly was whether the article explicitly mentioned data repositories, appendices, or other further information about the empirical database or methodological appendices. Again, we coded this criterion as “1” even if the authors explicitly stated that the information could not be shared due to data privacy concerns. Very few studies explicitly mentioned such concerns about confirmability. Given that such direct reporting on transparent or confirmable reporting is relatively rare, we also looked for cases where authors motivated their choice or described their methodological approach in detail. Here we found several examples, such as in Kokko and Lagerkvist (2017):

“All interviews were transcribed for analysis, which involved open coding of narrative descriptions according to the grounded theory generation procedure described by (...), and development of thematic categories and abstraction of conceptual metaphors to categories. The qualitative data organization software Atlas.ti Version 7.5.7 was used for organization of coding and categorization. When coding, especially in the construct elicitation step, the aim was to make broad enough categories of meaning for the elements of the ladders (A-C-V) in order to obtain links identified by more than one participant, without losing the relationships between the elements and not focusing on the elements themselves (…). Applicable codes were created for pair–construct relationships, such as health and sickness, save or spend money, and sustaining or difficulty in sustaining daily living, (…). (p. 211)”

Part of confirmability is therefore whether the articles justify the choice of the approach, the choice of empirical source, and the selection of observations (if applicable). Here, the articles provided much more detailed information (see second row of Figure 2). Most articles implicitly or explicitly discussed why a particular approach (“approach” in Figure 2) was used – for example, realist evaluation or contribution analysis. About half of the articles also justified the empirical sources (“choice”) and observations (“selection”). Although not all studies lend themselves to such discussions, it is interesting to learn why, for example, an evaluation relies mainly on interviews rather than focus groups, or why it selects certain types of stakeholders but not others. Sometimes a brief justification of the choice of methods is sufficient to help the reader:

“We also recognised that a consensus view of the programme needed to be achieved for things to progress. To do this, we decided to hold a participatory workshop drawing on the principles of a [...] strategic assessment approach.” (Siebert & Myles, 2019, p. 471).

There are few systematic differences in the reporting of confirmability across different designs or categories. In other words, it makes little difference whether we looked at an article that used stakeholder analysis, developmental evaluation, or both. When we look at the rationale given for specific choices, we find that few articles go into detail. Take the example of stakeholder analysis, the most common category identified above. While it is difficult to divide stakeholder analysis into different groups, qualitative evaluators may have different types of stakeholders in mind. In a participatory approach, stakeholders include all those affected by an intervention (target group), whereas an evaluator guided by the concept of veto players would focus only on stakeholders who are powerful or institutionally relevant actors (see e.g. Jepsen & Eskerod, 2009; Reed et al., 2009). In practice, we found few such discussions in the articles, but they might be helpful to understand more systematically what kind of stakeholder analysis was conducted.

A final criterion is the simple reporting of limitations. Many articles – instead of reporting explicit standards – explicitly deal with limitations, thus revealing potential weaknesses or shortcomings in a transparent way. In this sense, this regular practice is universal and includes all types of evaluation methods. Figure 2 shows that 60% of all studies discuss their own (methodological) limitations. In the remaining cases, it may make sense not to mention the limitations, but it may also be a missed opportunity.

### Limitations of the Study

Our analysis of the methods and reporting criteria thus leads to mixed results. On the one hand, we find a wide variety of methods and great examples of how to deal with the reporting criteria we selected. On the other hand, evaluation practice tends to concentrate on a few approaches and methods and to report only partially on the criteria. We also did not find large differences in reporting standards across methods and designs. One reason for this may be that the authors publish the articles in scientific journals to highlight points of interest that arise from the evaluation, but these are only excerpts from the underlying evaluations and their findings. Often, such information can be found in other documents, such as the detailed evaluation reports, to which we did not have access. Although we did our best to locate such documents where they were mentioned, we cannot really verify this in our analysis. While it is true that many evaluations take place outside the context of academic journals, these journals are still important portals to curated evidence bases for large and complex fields of inquiry. For this reason, we believe that these articles are highly relevant to our questions, and we believe that stricter adherence to reporting standards would benefit everyone.

Other difficulties with coding apply to our own analysis. We went through our codebook and our coding several times, and yet we may have missed passages in individual articles. We are also aware that our categorizations can be challenged. For example, it is debatable whether theoretical tools such as a “theory of change” are part of the same group as “stakeholder analysis” or “developmental evaluation”. The sources we have chosen for categorization, such as handbooks and textbooks, can be criticized. It will be interesting to see if other authors compile similar information to replicate our findings.

In addition, Liao and Hitchcock (2018) also note the possibility that journal editorial practices may lead to limited descriptions of procedures in evaluations, especially given space constraints. Indeed, we find some, albeit limited, variation for the five journals we examined (results available upon request). While our sample is too small to explore this hypothesis in more detail, we believe that variation in editorial policies could be an interesting area for further research.

Another limitation is the short time span of the articles. In future research, we plan to use a larger dataset, relying mainly on quantitative tools to investigate whether there are significant trends in reporting, also in relation to different methods. To reiterate, our small sample selection does not claim to be representative. The population from which a random sample should be drawn is not obvious, given the large number of publication outlets. Therefore, we have tried to cover a broad range of evaluation areas and to minimize our own bias. We plan to follow up our analysis with a more thorough quantitative content analysis of a larger set of evaluations. This may also allow us to find more nuanced differences in reporting standards across methods and designs. Nevertheless, our findings have some degree of transferability, both to the larger sample of 1070 articles and to other types of publications reporting evaluation findings.

## Conclusion

Our research is motivated by the question of how qualitative evaluations can increase the visibility and relevance of their results. A key measure is to follow reporting standards on evaluation design that allow their readers to assess the quality of the results. The diversity of qualitative evaluation approaches and methods, which are rooted in different ontologies and epistemologies, makes it difficult, but not impossible, to establish widely accepted reporting standards. Indeed, we found that most qualitative evaluations use relatively similar designs, methods and empirical sources. We therefore argue for stricter adherence to reporting reflexivity, confirmability and transferability as common standards, which would greatly facilitate the accumulation of knowledge across these studies.

The results of our study are transferable in two ways. First, the fact that many studies use similar designs is relevant to the debate on qualitative evaluation methods. Despite our initial suspicion of a vast - sometimes incommensurable - diversity, we find a degree of comparability and ultimately transferability between the articles we reviewed. Many studies use some form of case study design, look at stakeholders and use interviews, for instance. While there are still significant differences in these categories, this convergence means that, with more harmonized reporting standards, we can move towards knowledge accumulation without sacrificing the strengths of qualitative evaluation.

Patton (2015, pp. 559–560) calls for the triangulation of qualitative and quantitative data sources to increase the credibility of any type of evaluation. Our findings are consistent with this methodological debate, showing that the practice of triangulation would be greatly facilitated by reporting standards such as those presented in Table 1. These common standards do not require convergence or methodological hegemony on either side, but create an interface for discussion and communication of findings. In this way, qualitative evaluations could play a greater role in impact evaluation, provided they fully exploit the strength of their methodological diversity (Barnow et al., 2024). To date, however, qualitative evaluations have been underutilized in impact evaluations (e.g. Sharma Waddington et al., 2025).

Second, our findings on adherence to reporting standards are also transferable to the ongoing debate on the construction and assessment of systematic reviews that include qualitative evaluations. The recommendation of Walsh et al. (2020) cited at the beginning of this article not to include poorly reported studies cannot be implemented for many evaluation topics, as often few qualitative evaluations are included in the first place (Barnow et al., 2024). Our findings therefore contribute to the debate on how to improve the overall quality of qualitative systematic evaluation reviews (e.g. Carroll et al., 2012; Sharma Waddington et al., 2025; Walsh et al., 2020). We believe that the editors of the leading evaluation journals also have a crucial role to play in this regard, by reviewing their editorial policies and asking authors to adhere to them more closely.

Moreover, the methodological strength of qualitative evaluations justifies their use in providing evidence for policy learning. Maxwell (2020) reminds us that these methods are well suited to examining the programs, context, and process of public policy. These are precisely the areas in which Howlett (2012), among others, sees huge potential for policy learning. He emphasizes that real policy learning includes the political embedding and feasibility of programs, which he refers to as “deeper” learning. By incorporating context, qualitative evaluations enable such deeper policy learning. In a similar way, qualitative evaluations contribute to recent debates on policy learning in contexts of public value creation, in which public policy is assessed more fundamentally on the basis of values and legitimacy (e.g. Drew & Kim, 2025). Qualitative evaluations tend to be more sensitive to such deeper, partly normative, partly political questions. They should be an essential part of public management. Our contribution argues that qualitative evaluations can make these contributions to policy learning and public management if they build on their methodological strengths of diversity and make them visible through transparent reporting.

Further research should address the question of how qualitative evaluations can be given more weight in the generation of evidence for policy learning. The first question would be how qualitative research design, method selection, and reporting are related, as some designs may make certain reporting elements seem too obvious or too complex. We have some early evidence of this, but much more data is needed to better understand these relationships.

In addition, while we focus mainly on relatively abstract, research-oriented standards, more practical standards such as cost-efficiency or timeliness are also worth exploring further. This is crucial for the practical applicability of qualitative evaluations. Very abstract tools of appraisal will not resonate in practice, and overly simple ones will often not do justice to the complex design of qualitative evaluations. Building on this, assessment tools could be developed in the long term that are practically applicable and relevant to the consumers of evaluations (Pollard & Forss, 2023). We could then see whether there are trade-offs between the more abstract reporting standards we examine, such as credibility or transferability, and those of more practical considerations, such as cost-efficiency. By balancing practical and theoretical insights in reporting, qualitative evaluation evidence would have much more of the relevance it deserves for policy learning.

## Supplemental Material

Supplemetnal Material - Taking Stock of Qualitative Methods of Evaluation: A Study of Practices and Quality Criteria Supplemental Material for Taking Stock of Qualitative Methods of Evaluation: A Study of Practices and Quality Criteria by Thilo Bodenstein and Achim Kemmerling in Evaluation Review

## Notes

1. Positivist logic assumes that reality exists independently of the researcher. Therefore, researchers can employ a hypothetical-deductive logic of inquiry to detect empirical regularities (Bryman, 2008). Constructivist and interpretive approaches, on the other hand, assume that reality is constructed through social interaction and meaning-making by actors and is accessible to research through an interpretative-hermeneutic methodology (Pawson & Tilley, 1997, pp. 17–20; Schwartz-Shea & Yanow, 2012, pp. 30–32). Although there are important epistemological differences between constructivist and interpretive approaches (Schwartz-Shea & Yanow, 2012, p. 8), in evaluation contexts it is often hard to clearly distinguish between these two logics of inquiry.

2. See Macklin and Gullickson (2022) for a more comprehensive list on validity criteria.

3. We did not include qualitative evaluations from the COVID and post COVID years from 2020 onwards, as the COVID-19 pandemic had a significant impact on qualitative research due to various mitigation measures (Suadik, 2022) which may have resulted in bias.

4. Background interview with a professional evaluator at a major institution evaluating development projects. Name and details must remain confidential.
