# Supplementary material for: Taking Stock of Qualitative Methods of Evaluation: A Study of Practices and Quality Criteria
Source: Eval Rev. 2025 Aug 28;50(1):89–115. doi: 10.1177/0193841X251370426 (PMC12715029; doi:10.1177/0193841X251370426)
Supplement: Supplemetnal Material - Taking Stock of Qualitative Methods of Evaluation: A Study of Practices and Quality Criteria [file sj-pdf-1-erx-10.1177_0193841X251370426.pdf]

Appendices

Appendix 1. Table Five Major Journals.

	Number	Percentage
American Journal of Evaluation	163	15
Evaluation	140	13
Evaluation Review	85	8
Evaluation and Program Planning	552	52
The Canadian Journal of Program Evaluation	130	12
Total	1070	100

Appendix 2. Full Codebook.

**Domain**	**Standard**	**Category**	**Source**
	Evaluand type	Program evaluation	
		Project evaluation	
		Research on evaluation	
		Evaluation theory	
		Meta evaluation	
	Sector	Development	
		Education	
		Social & Labor	
		Economic	
		Health Care	
		Organization/Administration	
		Business	
		Infrastructure	
		NGO/Participation	
		Crime	
		Other	
**Investigation design and method**	Evaluation purpose	Exploration	CHESS (adapted)
		Causal	
		Descriptive	
		Interpretative	
	Evaluation approach	Stakeholder Analysis	CHESS

		Community<br>Realist Evaluation<br>Contribution Analysis<br>Participatory<br>Developmental evaluation<br>Process Tracing<br>Quantitative evaluation<br>Cost-Benefit-Analysis<br>Theory of change<br>Formal Theory<br>Network Analysis<br>Experiment	(adapted)
	Meta-theory	Rationalist<br>Constructivist<br>Interpretative<br>Other	
	Research design	Case Study<br>Longitudinal<br>Cross-sectional<br>PTCS<br>Mixed methods<br>Comparison<br>Meta-analysis<br>Other	CHESS<br>(adapted)
	Data collection instruments	Survey<br>Interviews<br>Focus groups<br>Documents/archives<br>Observation<br>Other interactive/ participatory method	CHESS<br>(adapted)
**People**	Affiliation	Academic<br>Professional (independent)<br>Business company<br>Government	CHESS<br>(adapted)

		Mixed	
	Evaluators' role	Author, evaluator and policymaker identical<br>Author is evaluator, but not involved in intervention<br>Author is not evaluator nor policymaker	CHESS (adapted)
**Quality criteria**	Reflexive stance	Background of author is discussed<br>Background is not discussed	
	Empirical and methodological limitations	Discussed<br>Not discussed	CHESS
	External validity/ generalizability	Mentioned<br>Not mentioned	
	Selection of observations	Discussed<br>Not discussed	CHESS (adapted)
	Choice of primary empirical source	Explained<br>Not explained	
	Data transparency/explicitness	Shown<br>Not shown	

Appendix 3. Table Inter-Coder Reliability.

	Percent Agreement	Krippendorff's Alpha	N Agreement vs. N Disagreement	N Cases vs. N Decisions
First Run (Deductive)	52.2%	0.453	24 vs. 22	46 vs. 92
Second Run (Deliberative)	96.4%	0.95	1059 vs. 39	1098 vs. 2196

Note: The IRR statistics are based on a selection of categories coded in each run. Due to a coding error in the first run, we used only a small sample of coded categories to code the IRR. In the second run, we sampled a much larger number of decisions to see if the settlements were actually higher (see Krippendorff 2011).

Appendix 4. Table Co-Occurrence Table of Evaluation Categories for 1070 Articles.

	Stakeholder		Community		Realist		Participatory		Developmental	
	**0**	**1**	**0**	**1**	**0**	**1**	**0**	**1**	**0**	**1**
**Stakeholder**										
0	937	0	771	166	906	31	900	37	911	26
1	0	133	96	37	126	7	114	19	129	4
**Community**										
0	771	96	867	0	834	33	831	36	844	23
1	166	37	0	203	198	5	183	20	196	7
**Realist**										
0	906	126	834	198	1.032	0	976	56	1.003	29
1	31	7	33	5	0	38	38	0	37	1
**Participatory**										
0	900	114	831	183	976	38	1.014	0	986	28
1	37	19	36	20	56	0	0	56	54	2
**Developmental**										
0	911	129	844	196	1.003	37	986	54	1.040	0
1	26	4	23	7	29	1	28	2	0	30

Appendix 5. Table Research Designs.

	1st codes		All codes	
	Frequency	Percent	Frequency	Percent
Case study	33	67	34	57
Longitudinal	2	4	4	7
Comparison	5	10	3	10
Mixed methods	4	8	8	13
Meta-analysis	4	8	5	8
Other	1	2	1	3
Total	49	100	60	100

Appendix 6. Table Main Type.

	1st code		All codes	
	Frequency	Percent	Frequency	Percent
Program evaluation	29	60	29	47
Project evaluation	11	23	12	19
Research on evaluation	4	8	16	26
Meta-evaluation	4	8	5	8
Total	48	100	62	100

Appendix 7. Table Metatheory.

		1st codes		All codes
Metatheory	Frequency	Percent	Frequency	Percent
Rationalist	41	82	44	73
Constructivist	3	6	6	10
Interpretative	4	8	8	13
Other	2	4	2	3
Total	50	100	60	100

Appendix 8. Table Major Aim.

		1st codes		All codes
	Frequency	Percent	Frequency	Percent
Exploration	19	38	22	28
Causality	17	34	28	35
Description	12	24	24	30
Interpretation	2	4	5	6
Total	50	100	79	100

Appendix 9. Articles in the Small Sample.

Adams, A. E., Nnawulezi, N. A., & Vandenberg, L. (2015). "Expectations to Ehange" (E2C): A participatory method for facilitating stakeholder engagement with evaluation findings. *American Journal of Evaluation*, *36*(2), 243–255. https://doi.org/10.1177/1098214014553787

Anderson, L. A., & Slonim, A. (2017). Perspectives on the strategic uses of concept mapping to address public health challenges. *Evaluation and Program Planning*, *60*, 194–201. https://doi.org/10.1016/j.evalprogplan.2016.08.011

Bamanyaki, P. A., & Holvoet, N. (2016). Integrating theory-based evaluation and process tracing in the evaluation of civil society gender budget initiatives. *Evaluation*, *22*(1), 72–90. https://doi.org/10.1177/1356389015623657

Campbell, R., Townsend, S. M., Shaw, J., Karim, N., & Markowitz, J. (2015). Can a workbook work? Examining whether a practitioner evaluation toolkit can promote instrumental use. *Evaluation and Program Planning*, *52*, 107–117. https://doi.org/10.1016/j.evalprogplan.2015.04.005

Caron, V., Bérubé, A., & Paquet, A. (2017). Implementation evaluation of early intensive behavioral intervention programs for children with autism spectrum disorders: A systematic review of studies in the last decade. *Evaluation and Program Planning*, *62*, 1–8. https://doi.org/10.1016/j.evalprogplan.2017.01.004

Chen, K. H.-J. (2017). Contextual influence on evaluation capacity building in a rapidly changing environment under new governmental policies. *Evaluation and Program Planning*, *65*, 1–11. https://doi.org/10.1016/j.evalprogplan.2017.06.001

Copestake, J., Allan, C., Bekkum, W. V., Belay, M., Goshu, T., Mvula, P., Remnant, F., Thomas, E., & Zerahun, Z. (2018). Managing relationships in qualitative impact evaluation of international development: QuIP choreography as a case study. *Evaluation*, *24*(2), 169–184. https://doi.org/10.1177/1356389018763243

Crooks, C. V., Exner-Cortens, D., Siebold, W., Moore, K., Grassgreen, L., Owen, P., Rausch, A., & Rosier, M. (2018). The role of relationships in collaborative partnership success: Lessons from the Alaska Fourth R project. *Evaluation and Program Planning*, *67*, 97–104. https://doi.org/10.1016/j.evalprogplan.2017.12.007

Dalkin, S., Lhussier, M., Williams, L., Burton, C. R., & Rycroft-Malone, J. (2018). Exploring the use of Soft Systems Methodology with realist approaches: A novel way to map programme complexity and develop and refine programme theory. *Evaluation*, *24*(1), 84–97. https://doi.org/10.1177/1356389017749036

David, P., & Schiff, M. (2015). Learning from bottom-up dissemination: Importing an evidence-based trauma intervention for infants and young children to Israel. *Evaluation and Program Planning*, *53*, 18–24. https://doi.org/10.1016/j.evalprogplan.2015.07.012

Downes, A., Novicki, E., & Howard, J. (2019). Using the contribution analysis approach to evaluate science impact: A case study of the National Institute for Occupational Safety

and Health. *American Journal of Evaluation*, *40*(2), 177–189. https://doi.org/10.1177/1098214018767

Frye, V., Paige, M. Q., Gordon, S., Matthews, D., Musgrave, G., Kornegay, M., Greene, E., Phelan, J. C., Koblin, B. A., & Taylor-Akutagawa, V. (2017). Developing a community-level anti-HIV/AIDS stigma and homophobia intervention in New York City: The project CHHANGE model. *Evaluation and Program Planning*, *63*, 45–53. https://doi.org/10.1016/j.evalprogplan.2017.03.004

Gosselin, J., Valiquette-Tessier, S.-C., Vandette, M.-P., & Romano, E. (2015). Evaluation of a youth agency's supervision practices: A mixed-method approach. *Evaluation and Program Planning*, *52*, 50–60. https://doi.org/10.1016/j.evalprogplan.2015.03.010

Ha, K.-M. (2019). Integrating the resources of Korean disaster management research via the Johari window. *Evaluation and Program Planning*, *77*, 101724. https://doi.org/10.1016/j.evalprogplan.2019.101724

Haarich, S. N. (2018). Building a new tool to evaluate networks and multi-stakeholder governance systems. *Evaluation*, *24*(2), 202–219. https://doi.org/10.1177/1356389018765797

Harper, L. M., & Dickson, R. (2019). Using developmental evaluation principles to build capacity for knowledge mobilisation in health and social care. *Evaluation*, *25*(3), 330–348. https://doi.org/10.1177/1356389019840058

Harris, K., Henderson, S., & Wink, B. (2019). Mobilising Q methodology within a realist evaluation: Lessons from an empirical study. *Evaluation*, *25*(4), 430–448. https://doi.org/10.1177/1356389019841645

Janssens, F. J. G., & Ehren, M. C. M. (2016). Toward a model of school inspections in a polycentric system. *Evaluation and Program Planning*, *56*, 88–98. https://doi.org/10.1016/j.evalprogplan.2016.03.012

Jiménez-Herranz, B., Manrique-Arribas, J. C., López-Pastor, V. M., & García-Bengoechea, E. (2016). Transforming a municipal school sports programme through a critical communicative methodology: The role of the of advisory committee. *Evaluation and Program Planning*, *58*, 106–115. https://doi.org/10.1016/j.evalprogplan.2016.06.003

Jones, M., Verity, F., Warin, M., Ratcliffe, J., Cobiac, L., Swinburn, B., & Cargo, M. (2016). OPALesence: Epistemological pluralism in the evaluation of a systems-wide childhood obesity prevention program. *Evaluation*, *22*(1), 29–48. https://doi.org/10.1177/1356389015623142

Kokko, S., & Lagerkvist, C. J. (2017). Using Zaltman metaphor elicitation technique to map beneficiaries' experiences and values: A case example from the sanitation sector. *American Journal of Evaluation*, *38*(2), 205–225. https://doi.org/10.1177/1098214016649054

Koleros, A., Jupp, D., Kirwan, S., Pradhan, M. S., Pradhan, P. K., Seddon, D., & Tumbahangfe, A. (2016). Methodological considerations in evaluating long-term systems change: A case study from eastern Nepal. *American Journal of Evaluation*, *37*(3), 364–380. https://doi.org/10.1177/1098214015615231

Koper, C. S., Lum, C., & Hibdon, J. (2015). The uses and impacts of mobile computing technology in hot spots policing. *Evaluation Review*, *39*(6), 587–624. https://doi.org/10.1177/0193841X16634482

Lawrence, R. B., Rallis, S. F., Davis, L. C., & Harrington, K. (2018). Developmental evaluation: Bridging the gaps between proposal, program, and practice. *Evaluation*, *24*(1), 69–83. https://doi.org/10.1177/1356389017749276

Leenstra, M. (2018). The human factor in development cooperation: An effective way to deal with unintended effects. *Evaluation and Program Planning*, *68*, 218–224. https://doi.org/10.1016/j.evalprogplan.2017.09.008

Lennie, J., Tacchi, J., Wilmore, M., & Koirala, B. (2015). A holistic, learning-centred approach to building evaluation capacity in development organizations. *Evaluation*, *21*(3), 325–343. https://doi.org/10.1177/1356389015590219

Martinaitis, Ž., Christenko, A., & Kraučiūnienė, L. (2019). Evaluation systems: How do they frame, generate and use evidence? *Evaluation*, *25*(1), 46–61. https://doi.org/10.1177/13563890188021

McIsaac, J.-L. D., Mumtaz, Z., Veugelers, P. J., & Kirk, S. F. L. (2015). Providing context to the implementation of health promoting schools: A case study. *Evaluation and Program Planning*, *53*, 65–71. https://doi.org/10.1016/j.evalprogplan.2015.08.003

Millett, L. S., Ben-David, V., Jonson-Reid, M., Echele, G., Moussette, P., & Atkins, V. (2016). Understanding change among multi-problem families: Learnings from a formative program assessment. *Evaluation and Program Planning*, *58*, 176–183. https://doi.org/10.1016/j.evalprogplan.2016.06.010

Milley, P., Szijarto, B., Svensson, K., & Cousins, J. B. (2018). The evaluation of social innovation: A review and integration of the current empirical knowledge base. *Evaluation*, *24*(2), 237–258. https://doi.org/10.1177/13563890187632

Mohammad, T., Azman, A., & Anderstone, B. (2019). The global three: A Malaysian lens on the challenges and opportunities facing restorative justice planning and implementation. *Evaluation and Program Planning*, *72*, 1–7. https://doi.org/10.1016/j.evalprogplan.2018.09.007

Morgan, N. R., Davis, K. D., Richardson, C., & Perkins, D. F. (2018). Common components analysis: An adapted approach for evaluating programs. *Evaluation and Program Planning*, *67*, 1–9. https://doi.org/10.1016/j.evalprogplan.2017.10.009

Najafizada, S. A. M., Labonté, R., & Bourgeault, I. L. (2017). Stakeholder's perspective: Sustainability of a community health worker program in Afghanistan. *Evaluation and Program Planning*, *60*, 123–129. https://doi.org/10.1016/j.evalprogplan.2016.11.004

Nielsen, J. V., Bredahl, T. V. G., Bugge, A., Klakk, H., & Skovgaard, T. (2019). Implementation of a successful long-term school based physical education intervention: Exploring provider and programme characteristics. *Evaluation and Program Planning*, *76*, 101674. https://doi.org/10.1016/j.evalprogplan.2019.101674

Nishimura, S. T., Hishinuma, E. S., Goebert, D. A., Onoye, J. M. M., & Sugimoto-Matsuda, J. J. (2018). A model for evaluating academic research centers: Case study of the

Asian/Pacific Islander Youth Violence Prevention Center. *Evaluation and Program Planning*, *66*, 174–182. https://doi.org/10.1016/j.evalprogplan.2017.02.010

Noordegraaf, M., Douglas, S., Bos, A., & Klem, W. (2017). How to evaluate the governance of transboundary problems? Assessing a national counterterrorism strategy. *Evaluation*, *23*(4), 389–406. https://doi.org/10.1177/1356389017733340

Norton, S., Milat, A., Edwards, B., & Giffin, M. (2016). Narrative review of strategies by organizations for building evaluation capacity. *Evaluation and Program Planning*, *58*, 1–19. https://doi.org/10.1016/j.evalprogplan.2016.04.004

Paradis, C. (2016). Canada's National Alcohol Strategy: It's time to assess progress. *Canadian Journal of Program Evaluation*, *31*(2), 232–241. https://doi.org/DOI: 10.3138/cjpe.276

Pouw, N., Dietz, T., Belemvire, A., de Groot, D., Millar, D., Obeng, F., Rijneveld, W., Van der Geest, K., Vlaminck, Z., & Zaal, F. (2017). Participatory assessment of development interventions: Lessons learned from a new evaluation methodology in Ghana and Burkina Faso. *American Journal of Evaluation*, *38*(1), 47–59. https://doi.org/10.1177/1098214016641210

Reeve, C., Humphreys, J., & Wakerman, J. (2015). A comprehensive health service evaluation and monitoring framework. *Evaluation and Program Planning*, *53*, 91–98. https://doi.org/10.1016/j.evalprogplan.2015.08.006

Richard, L., Fortin-Pellerin, L., Chiocchio, F., Litvak, É., Champagne, F., & Beaudet, N. (2016). Création de connaissances organisationnelles à la suite d'une intervention de développement professionnel en Centre de santé et de services sociaux (CSSS): Une évaluation des laboratoires de promotion de la santé. *Canadian Journal of Program Evaluation*, *31*(2), 184–210. https://doi.org/10.3138/cjpe.343

Rolfe, S. (2019). Combining theories of change and realist evaluation in practice: Lessons from a research on evaluation study. *Evaluation*, *25*(3), 294–316. https://doi.org/10.1177/1356389019835229

Shmueli, D. F., Ben Gal, M., Segal, E., Reichman, A., & Feitelson, E. (2019). How can regulatory systems be assessed? The case of earthquake preparedness in Israel. *Evaluation*, *25*(1), 80–98. https://doi.org/10.1177/1356389018803235

Siebert, P., & Myles, P. (2019). Eliciting and reconstructing programme theory: An exercise in translating theory into practice. *Evaluation*, *25*(4), 469–476. https://doi.org/10.1177/1356389019870211

Sokol, R., Moracco, B., Nelson, S., Rushing, J., Singletary, T., Stanley, K., & Stein, A. (2017). How local health departments work towards health equity. *Evaluation and Program Planning*, *65*, 117–123. https://doi.org/10.1016/j.evalprogplan.2017.08.002

Soura, B. D., Bastien, R., & Fallu, J.-S. (2016). Étude d'évaluabilité d'une intervention visant à prévenir l'usage de substances psychoactives lors de la transition primaire-secondaire. *Canadian Journal of Program Evaluation*, *31*(2), 211–231. https://doi.org/10.3138/cjpe.304

Sturges, K. M. (2015). Complicity revisited: Balancing stakeholder input and roles in evaluation use. *American Journal of Evaluation*, *36*(4), 461–469. https://doi.org/10.1177/1098214015583329

Suiter, S. V. (2017). Community health needs assessment and action planning in seven Dominican bateyes. *Evaluation and Program Planning*, *60*, 103–111. https://doi.org/10.1016/j.evalprogplan.2016.10.011

Visser, M., Thurman, T. R., Spyrelis, A., Taylor, T. M., Nice, J. K., & Finestone, M. (2018). Development and formative evaluation of a family-centred adolescent HIV prevention programme in South Africa. *Evaluation and Program Planning*, *68*, 124–134. https://doi.org/10.1016/j.evalprogplan.2018.03.002
